# 

**Published:** 1878-06-20

**Authors:** 


☞All communications relating to the business of THE RECORD for the years 1877 and 1878, must be addressed to DR. R. C. WORD, Managing Editor Southern Medical Record, Atlanta, Ga.

☞Brief and practical communications are solicited on all subjects pertaining to medicine; also reports of cases in practice.

☞Send money by check, postal order or registered letter.

☞Write your name, post-office, county and State plainly.

THE IMAGE OF HER MOTHER.

A NOVEL.

BY RUTH RUSTIC.

In the Savannah Weekly News, 20th April, was commenced a new serial story, with the above title, written by a lady of Savannah. The Weekly News is the largest and best weekly in the South. It is a complete newspaper, and contains the latest telegraphic and State news, markets, etc., an agricultural and military department. It is adapted for general circulation throughout the South. Subscription, one year, $2.00; six months, $1.00. Specimen copies sent free. Address

J. H. ESTILL,
Savannah, Ga.

---

Receipted—1878: L. S. Brownlee, Thos. S. Jones, E. L. Smith, H. H. Malone, R. Blacknall, R. C. Runner, P. J. Fonts, W. B. Thomason, D. H. Connelly, Blue & Turner, L. P. Hamner, S. H. Anderson, J. M. L. Alford, H. R. Thorpe, J. L. Phelps, F. M. Fitzhugh.

